# Rapidly Measuring the Speed of Unconscious Learning: Amnesics Learn Quickly and Happy People Slowly

**DOI:** 10.1371/journal.pone.0033400

**Published:** 2012-03-23

**Authors:** Zoltan Dienes, Roland J. Baddeley, Ashok Jansari

**Affiliations:** 1 School of Psychology, University of Sussex, Brighton, United Kingdom; 2 School of Experimental Psychology, University of Bristol, Bristol, United Kingdom; 3 School of Psychology, University of East London, London, United Kingdom; Institute of Psychiatry at the Federal University of Rio de Janeiro, Brazil

## Abstract

**Background:**

We introduce a method for quickly determining the rate of implicit learning.

**Methodology/Principal Findings:**

The task involves making a binary prediction for a probabilistic sequence over 10 minutes; from this it is possible to determine the influence of events of a different number of trials in the past on the current decision. This profile directly reflects the learning rate parameter of a large class of learning algorithms including the delta and Rescorla-Wagner rules. To illustrate the use of the method, we compare a person with amnesia with normal controls and we compare people with induced happy and sad moods.

**Conclusions/Significance:**

Learning on the task is likely both associative and implicit. We argue theoretically and demonstrate empirically that both amnesia and also transient negative moods can be associated with an especially large learning rate: People with amnesia can learn quickly and happy people slowly.

## Introduction

The process by which we can incidentally acquire knowledge of the structure of the environment without being aware of the knowledge is called implicit learning [1], [2]. Implicit learning is a fundamental process involved in mastering music, languages, social and cultural rules, perceptual-motor skills, and almost any domain involving the gradual refinement of judgment or action [3], [4]. Implicit learning is normally investigated by requiring people to learn complex structures, like finite state grammars or complex control systems [5]. Such tasks are ill-suited for determining an individual’s effective learning rate, because a neural network or other model will typically have an optimal learning rate in the middle of its range with either a very high or very low learning rate producing slower learning overall for the system on such complex tasks [6]. Perhaps for this reason, researchers have not systematically addressed the question of what factors influence implicit learning rate per se, despite the fundamental nature of the question (though cf [7] outside the context of implicit learning paradigms).

We introduce a task to measure the learning rate of a person quickly and simply. A fast or large learning rate means, by definition, that each trial changes strength of prediction by a large amount, and thus recent trials will have a large influence on the current prediction. Consequently, more distant trials will have a relatively smaller influence. Conversely, a small (slow) learning rate means, by definition, that each trial introduces a small change to strength of predictions, prior knowledge is changed only marginally, and distant trials will have a relatively strong influence on current predictions. Thus, for example, on a simple conditioning task, a large learning rate corresponds to learning the simple association quickly.

In our task, the participant makes a series of binary predictions, e.g. whether a probabilistic stimulus will appear on the left or the right. Our sequences were all in fact random. Despite the random nature of the sequence, a learning device will on any given trial be influenced by the idiosyncratic pattern of past trials to have an expectation of right or left. We correlate what the participant predicts on a trial with what happened one trial back, two trials back, etc. The random nature of the sequence means each of these correlations is independent. That is, each correlation directly indicates the influence of events a given number of steps in the past on current predictions regardless of what happened on any other number of steps into the past. If a manipulation increases learning rate it will show in the plot of correlations against number of trials into the past: Correlations of the current prediction with recent trials will increase and correlations of the current prediction with distant trials will decrease. The simplicity of the task is what allows it to be a tool for clearly measuring learning rate.

In the first two experiments we demonstrate relevant properties of our method as a measure of implicit learning rate, namely that it involves associative learning rather than simply conceptual priming, and it also involves the phenomenology of guessing, characteristic of implicit learning. In the second two experiments we motivate the method by illustrating its use in particular domains, showing how it sheds light on amnesia and also on the way emotional stimuli influence learning. Knowing learning rates can allow surprising conclusions in a range of psychological domains.

## Experiment 1

Experiment 1 explored whether the learning on the task was associative or involved just conceptual priming of “left” or “right”. We tested the associative nature of learning on the task by having a distinctive context (a tone) associated with most trials, but absent on every fourth trial. In classical conditioning, more salient stimuli acquire more associative strength than less salient stimuli, a phenomenon called over-shadowing [8]. Less salient stimuli may acquire very little associative strength because of the presence of a salient stimulus. Thus, if people are learning to associate contextual cues with the prediction for left or right, removing salient cues should reduce the reliance of the prediction on past trials. That is, if associative learning has occurred, then there should be a weaker dependency of predictions on past trials for no tone trials than for tone trials.

### Methods

#### Participants

Thirty-four students from the University of Sussex participated. The protocol used in this and subsequent experiments was approved by the University of Sussex School of Psychology Research Governance Committee following the guidelines for human research of the British Psychological Society. Written informed consent was obtained from all participants in all experiments.

#### Procedure

On each trial the word “ready” was first displayed for 400 ms. On tone trials, there was a simultaneous 500 Hz tone; ‘ready’ was displayed in white Sanserif Turbopascal size 8 font. By contrast, every fourth trial had no tone (that is, there were always three tone trials between every no-tone trial), and ‘ready’ was displayed in yellow Gothic size 4 font. Participants were then instructed to press Z or M to predict left or right respectively, which they did in their own time. A square was displayed randomly on the left or right for 400 ms; on tone trials it was coloured blue and on no tone trials it was yellow. Finally there was a wait of 800 ms to make sure the tone of the next trial was heard as a warning for that trial and not a response to the previous one. There were 300 trials in total. Each participant experienced a different random sequence.

### Results and Discussion

**Figure 1:**
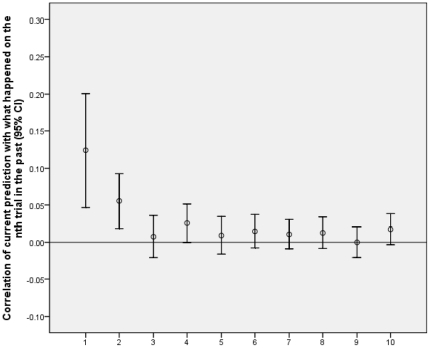
Results for testing whether learning is associative. Correlation of current prediction with what happened on the nth trial in the past plotted against trials into the past (n). Figure 1 shows the data for tone trials

**Figure 2:**
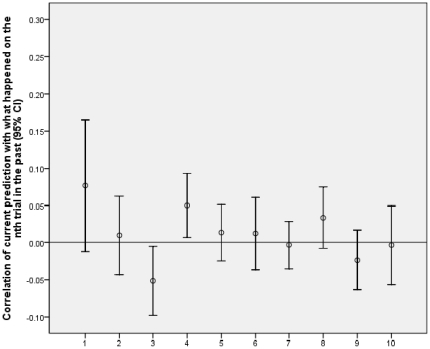
Results for testing whether learning is associative. Correlation of current prediction with what happened on the nth trial in the past plotted against trials into the past (n). Figure 2 shows no tone trials. Figure 2 is based on fewer trials than 1, hence the wider confidence intervals. The overall level of correlation is detectably stronger for Figure 1 rather than 2, indicating context is important

In experiment 1 participants were presented with a majority of trials involving a tone accompanying the ready prompt and in some trials the tone was missing. Figures 1 and 2 show the Pearson (i.e. phi) correlation of current prediction with where the square actually was from one to ten trials back for experiment 1. For example, for one trial back, the correlation shows how strongly what happened on the just preceding trial influenced the prediction on the current trial. Note that this is not the influence of the subject’s prediction in the previous trial on the current prediction - but the influence of where the square actually just was in the previous trial on the current prediction. If the learning rate was one, a single trial would result in maximum associative strength for whatever just happened, and so prediction would correlate one with the event one trial back. With a learning rate of one, if the square had been on the right on the previous trial, the subject would predict right 100% of the time on the next trial (and the same for left). Thus, of necessity, the correlation with all trials more than one trial back would be zero. On the other hand, if the learning rate was less than one, the correlation with events one trial back would be less than one, and events further back could influence prediction. Thus, the profile of influence over time gives information about learning rate. Further, only with a learning rate of zero would there be no influence on any trial; thus a general influence from the trials overall indicates learning occurred.

Figure 1 shows the data for tone trials and Figure 2 for no tone trials. The average correlation of predictions with what happened for trials one to ten back was detectably higher for tone trials (.03, SD = .04) than no tone trials (.01, SD = .07), t(33) = 2.03, p = .05, dz = 0.35, supporting the claim that learning in the task is associative. On the tone trials, overall the correlations were above chance, t(33) = 3.77, p = .001, 95% CI [.013, .043], showing that overall people were influenced by past trials; conversely, for the no tone trials the correlations were not on average detectably above chance, t(33) = .96, p = .35, 95% CI [–.013, .036]. While the latter result may be due to the larger standard errors for the no tone trials, the difference between tone and no trials cannot be due to larger standard errors in the latter, as the difference is significant. In sum, people’s learning was influenced by context with evidence of learning particularly when the context was relatively common. If learning on the task consisted merely of non-associative priming of the abstract concepts of left and right (i.e. if seeing something on the left primed a tendency to respond left regardless of context) then the presence or not of a tone would be irrelevant. Associative learning predicts an influence of context, as we found.

The results do not illuminate the basis of the associative learning, for example whether based on exemplar coding of a whole trial (e.g. tone plus side of square) (cf [9]) rather than a strength-based mechanism (like Rescorla Wagner). We can be sure that whatever the context-linking mechanism, however, it involves developing sensitivity to several trials in the past, so is not based on a memory of just one trial back (contrast [10]).

[11] and [12] used a similar task as ours, but with a reaction time measure of learning. Participants had to press a button when a stimulus appeared, which could be indicated by a tone. [12] argued (contrary to [11]) that rather than associative learning, *response* priming could account for the RT benefits, i.e. people press a button faster when they have just pressed it. While this is a possible explanation in their task, in the current task subjects make a prediction about a random stimulus so repeating the response of the previous trial would not produce any sensitivity of the current response to the stimulus location on the previous trial. Yet what we show is strong sensitivity of the current response to what the *stimulus* was on the just previous trial. Thus, this sensitivity cannot be response priming.

A weakness of the method is illustrated by the negative correlation three time steps back. Associative learning would only produce positive correlations. [13] found that when people were asked to predict a binary event (presence or absence of an air puff), such predictions were susceptible to the gambler’s fallacy, even when an eye-blink response showed standard conditioning. That is, the more often an air puff followed a tone, the less subjects expected it after a tone. Figure 1 illustrates that for the conditions of our method (including the rapid time scales: [13] had an interval of 10 seconds between trials instead of less than a second we used), the gambler’s fallacy is swamped by a process producing positive correlations, except for three trials back. Nonetheless, the contribution of the gambler’s fallacy to our findings is an important issue which we consider again in the discussion, with additional analyses.

Indeed, an alternative interpretation of the difference between tone and no trials is that the diminished sensitivity to past trials in the latter case reflects not diminished associative learning but an enhanced gambler’s fallacy tendency on no tone versus tone trials. However, a change in context has been found to reduce the tendency to use the gambler’s fallacy (with a coin toss) [14], rendering gambler’s fallacy a less plausible explanation of experiment 1.

As the structure to be learned is minimal (i.e. the sequence is random) people are learning spurious correlations when they become sensitive to past trials. In order to show that learning does occur on the task when there is real long term structure, 60 subjects were ran on the task (all trials no tones) where the probability systematically changed over trials. Specifically, for half the participants, for the first 120 trials the probability of left was 60%, then for 40 trials it was 50%; then for 120 trials it was 40%; for the other half of the participants the blocks occurred in reverse order. While people did not probability match perfectly, there was a 9% change in the actual probability of the stimulus occurring on the left or right in the final rather than first block, the probability of people’s responses changed by 6% in the appropriate direction, t(59) = 4.09, p<.0005, illustrating that people do learn structure in the task.

## Experiment 2

In experiment 2 we sought to determine the implicit nature of the knowledge. Implicit learning is a process by which people acquire knowledge of the structure of an environment without being aware of what that knowledge is [15]. In addition, implicit learning involves not only unconscious structural knowledge but also on occasion, produced by that structural knowledge, expectations which people are unaware of having [16]–[18]. Thus, in experiment 2 we determined if people were aware of having any knowledge.

### Methods

#### Participants

Fifty participants were recruited from the University of Sussex students and alumni to obtain a range of ages (20 to 67) so that any effect of age on the task could be controlled for when investigating amnesia below. These participants are the normal controls for the amnesic patient in experiment 3, and discussed further below.

#### Procedure

The timings were the same as in experiment 1. After ‘ready’ was displayed for 400 ms (no tone was sounded), participants were instructed to press the X key if they purely guessed left; Z if they had any confidence in a left prediction; N if they purely guess right; and M if they had any confidence in their right prediction. Participants were told that despite the fact that the sequence was random they may develop expectations of left or right; if they are aware of any expectations they should indicate some confidence.

### Results and Discussion

Experiment 2 asked people about their phenomenology. If people are sometimes unaware of expectations people should be influenced by past trials when they believe they are purely guessing. In experiment 2, people said they were guessing on 66% (SD = 24.5%) of trials. On those trials, predictions were influenced by past trials, the average correlation of predictions with what happened for trials one to ten back was .02 (SD = .04), significantly above zero, t(49) = 2.64, p = .011, d = 0.37. On trials in which people were sure, the average correlation was .00 (SD = .08).

In the applications to particular domains below we will distinguish between recent and distant influences on current predictions. To provide a measure of recent influences, the correlations of current prediction with past occurrences of the square for one and two trials back were averaged together. To provide a measure of distant influences, the correlations for four to ten trials into the past were averaged together. The division is based on the predominance of the gambler’s fallacy at three trials back only. We use the same division in the remaining experiments.

When people said they were guessing, the mean level of recent influence was .07 (SE = .018), significantly different from zero, t(47) = 3.88, p<.0005, d = 0.55, and the mean level of distant influence was .01 (SE = .005), significantly different from zero t(49) = 2.17, p = .035, d = .31. Thus, people showed significant learning of both recent and distant events even when they thought they were purely guessing. The corresponding figures for when people were partially sure for recent and distant influences were .06 (.035) and .00 (.01).

Experiment 4 provided further data on people’s learning when they believed they had used guessing or intuition, using different stimuli than experiment 2. In Experiment 4 it is argued that mood will affect learning, and either a happy or a sad mood was induced. Only the sad condition is analysed here because the happy condition eliminated any clear signs of learning overall. Participants reported guessing or using intuition on 65% (SD = 25.3%) of trials indicating the phenomenology characteristic of implicit learning on a majority of trials. On those trials, predictions were influenced by past trials, the average correlation of predictions with what happened for trials one to ten back was .02 (SD = .03), significantly above zero, t(26) = 2.77, d = 0.53, p = .01. On trials in which people were using rules or recollections, the average correlation was .00 (SD = .10).

In sum, a majority of trials involved a phenomenology characteristic of implicit learning, i.e. feelings of guessing or of intuition, while demonstrating sensitivity to structure. Having established that the method does measure the rate of specifically implicit learning, we turn now to consider two applications of the method.

## Experiment 3

In experiment 3 we applied the method to understanding amnesia. People with anterograde amnesia, following damage to the temporal lobes and underlying regions, have difficulty creating new explicit long-term memories resulting in major impairments in recalling post-morbid events. Nonetheless, they can be near normal in acquiring procedural skills [19]. A standard explanation is that there are different memory systems, for example a procedural one and also an episodic or declarative one, and people with anterograde amnesia have damage only to the latter [20]. Another explanation is that damage to the temporal lobes changes the learning rate of a single system (or at least of a relevant system). Two possibilities are that amnesia is a result of (1) an increase or (2) a decrease in learning rate compared to normal. We consider each possibility in turn.

Perhaps people with amnesia have a small learning rate on all tasks [21]–[23]. Thus, they cannot perform one-shot learning like storing one-off episodes in their life, but they can still fine tune procedural skills over many trials, which requires a small learning rate. Shanks and his colleagues have simulated learning in complex tasks and fitted the data by assigning people with amnesia a lower learning rate than healthy controls. This evidence is suggestive but depends on complex tasks where performance can be made worse by either an increase or a decrease in learning rate from its optimal value. Here we explore the relation between amnesia and learning rate using our method where there is a more transparent relation between data and learning rate.

While the hypothesis that amnesia is associated with as small learning rate seems plausible, it has some counter-intuitive consequences. If amnesia is associated with a smaller learning rate, each new trial makes a small contribution to associative strength, so associative strength depends on a proportionately greater influence of past trials. In that sense, learning rate is a measure of memory into the past: The smaller the learning rate, the longer the memory into the past. On these grounds one might expect that amnesia is associated with a large learning rate: Responses depend mainly on only the last trial or two and hence memory into the past is short. For example, with a learning rate of 1, current predictions would depend completely on the one previous trial and memory would go only one time step into the past.

It is likely people adjust learning rates to different tasks. In a task with the random structure we used, there is no “optimal” learning rate: all strategies will lead to the same performance. In a slowly changing world, small learning rates will average out the noise by taking into account many trials, and in a quickly changing world, a faster learning arte will more effectively track these changes. Thus we expect normal people to have relatively small learning rates on our task. The question is whether amnesic people will have a small learning rate on this task also.

### Methods

#### Participants

JC is discussed in chapter 4 of [24], referred to as case “Jay”. JC suffered an aneurysm at the age of 20, resulting in severe anterograde with virtually non-existent retrograde amnesia. An MRI scan indicates lesions are restricted to the hippocampal area. On the Wechsler Memory Scale, immediate recall is 8 (normal) and delayed recall is 0 (severely impaired). He is severely impaired also on the Rivermead Behavioural Memory Test and recall of the Rey-Osterreith Complex Figure. By contrast, recall of childhood and (pre-morbid) early adult life is normal.

At the time of testing JC was 42 years old. We recruited 30 University of Sussex students and alumni to create an even spread of ages from around 20 s to 60 s; mean age was 40.1 years (SD = 15.8), range 20 to 67. The correlation of recent influences with age was .05, not detectably different from zero, 95% CI [–.40, .32]. The correlation of distant influences with age was –.26, also not detectably different from zero, 95% CI [–.57, .11]. To increase power for comparing with JC, this sample was combined with another of 20 Sussex students, aged in their 20 s, and the combined sample of 50 students and alumni used as the controls for JC. Note that as JC was a University student when he suffered his aneurysm, all controls were University educated.

#### Procedure

The same procedure as experiment 2 was used. JC performed the task on 18 separate days, for 300 trials on each day. Each control participant performed the task once, for 300 trials.

### Results and Discussion

**Figure 3:**
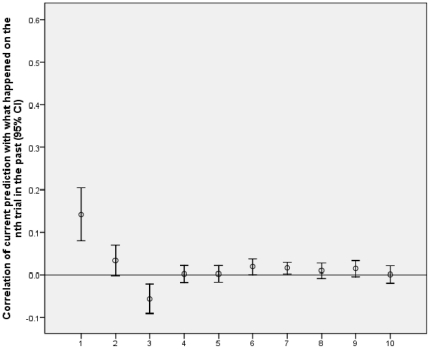
Results for testing normal controls. Correlation of current prediction with what happened on the nth trial in the past plotted against trials into the past (n).

**Figure 4:**
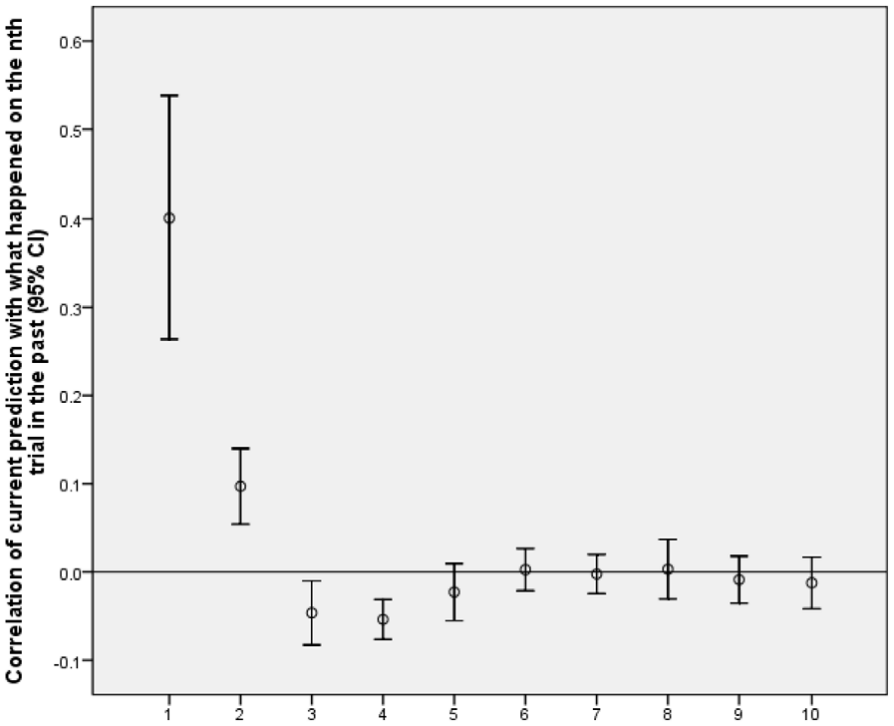
Results for testing JC. Correlation of current prediction with what happened on the nth trial in the past plotted against trials into the past (n). JC shows a stronger influence of recent trials than normal controls, and normal controls show a stronger influence of distant trials than JC.

Experiment 3 tested a dense amnesiac, JC, and matched controls. Figures 3 and 4 show the profile of correlations of current predictions with what happened for from one to ten trials into the past for JC and the normal controls in experiment 3. The pattern is consistent with JC having a large rather than small learning rate. The strength of recent influences (as defined above) was stronger for JC (.25, SD = .13) than for controls (.09, SD = .13), t(66) = 4.56, p<.0005, d = 1.24, while the strength of distant influences (as defined above) was stronger for controls (.01, SD = .03) than for JC (–.01, SD = .02), t(66) = 2.73, p = .008, d = 0.79. The data provide impressive support for the theory that people with amnesia, at least on this task, have an exceptionally high learning rate, and against the theory that people with amnesia have a generalised low learning rate. Note the evidence applies separately for recent and distant trials: One cannot try to e.g. explain away the evidence just for recent trials because the distant trials also provide evidence that JC has an especially large learning rate (and vice versa).

Other paradigms have found a range of results for the rate of conditioning for people with amnesia. For example, people with amnesia can show slower trace eye blink conditioning compared to controls [25], and equivalent delay eye blink conditioning [26]. Thus, we do not claim that people with amnesia have a generalised large learning rate, nor that a single system explains human learning (e.g. see [27] and [28] for dual systems approaches to learning in general). Our working hypothesis is that people normally do not have a fixed learning rate, but adjust according to how slowly the world appears to be changing and the amount of noise that needs to be averaged out. In a noisy slowly changing world small learning rates are optimal because they average the noise out of as many trials as possible. In normal people, learning may proceed by selecting from multiple learning devices for the one with the most effective learning rate for the task at hand (cf [7]). Thus, normals have a relatively low learning rate on the current task (random probability structure static over many trials), but a relatively large learning over a few conditioning trials with a clear signal. People with amnesia may be more rigid in the learning rate they can settle on. The task we have introduced provides a simple environment in which such issues can be explored.

## Experiment 4

In experiment 4 we applied the method to understanding the role of emotion in learning. According to the “affect as information” hypothesis [29]–[30] mild transient affective feelings arising with the performance of a task may be experienced as feedback about one’s performance. Success feedback should lead to use of prior knowledge and failure feedback to learning [31]–[32]. For example, people surreptitiously induced to be sad rather than happy rely more on stereotypes in social judgments. If the “affect as information” argument applies to implicit learning, then a surreptitious induction of a sad mood should indicate to the learning system that what it knows is not working: It can’t rely on prior knowledge but needs to attend to the present. That is a sad rather than happy mood should be associated with a larger learning rate. Similarly, according to the theory, happy moods lead to more global processing and more integration [33]; i.e. happy moods should lead to a small learning rate, involving a greater integration of information over time.

### Methods

#### Participants

Twenty-seven participants from the University of Sussex participated in the sad condition and 29 in the happy.

#### Procedure

In this experiment the random stimulus to be predicted was a face, which appeared on the left or right of the screen. The same timings were used as in the previous experiments on half the trials. On these trials an emotionally neutral male or female face was used as the stimulus. The remaining half of the trials (randomly intermixed with the neutral trials) were the mood inducing trials. The neutral trials were inserted to decrease habituation to the mood inducing stimuli. On the mood trials, the word ‘ready’ was displayed for 200 ms, the word ‘sad’ or ‘happy’ (depending on group) was displayed for 100 ms, then the word ‘ready’ was displayed for 200 ms (thus, the word ‘ready’ was displayed for 400 ms altogether, as in the previous experiments). The neutral face was displayed (on the left or right) for 200 ms, then either a sad or happy face (depending on group) was displayed for 150 ms, and the neutral face again for 200 ms. All faces were equally likely to be male or female.

On all trials, participants were instructed to press the X key if they chose left because they were purely guessing or using intuition; Z if they using some conscious rule or recollection of a pattern to predict it will appear on the left; N for choosing right on the basis of guessing or intuition; and M for choosing right on the basis of a rule or recollection.

At the end of the experiment participants rated on a 1 to10 scale how happy, sad, and alert they felt.

### Results and Discussion

In experiment 4, we investigated the effect of mood on learning rate. Mood valence was measured by subtracting the sad rating from the happy rating. Participants in the happy condition had a more positive valence (3.1, SD = 3.0) than those in the sad condition (1.0, SD = 3.0), t(50) = 2.58, p = .013, d = 0.72. Usefully, participants in the happy condition were not detectably different in alertness (4.2, SD = 2.0) than those in the sad condition (4.3, SD = 2.2), t(50) = .10, p = .92, d = .02, 95% CI [–1.2, 0.7], consistent with the manipulation changing only the valence and not the arousal of participants’ mood.

The mean recent influence in the sad condition (.13, SD = .11) was, as predicted, greater than that in the happy condition (.03, SD = .16), t(52) = 2.54, p = .014, d = .70. The mean distant influence was not detectably different between sad (–.00, SD = .02) and happy conditions (.00, SD = .02), t(53) = 0.64, p = .47, d = .20, 95% CI on the difference [–.01, +.01]. A Bayes factor (see Materials and Methods for explanation) indicated the data for recent influences provided, relative to the null, strong support for the theory the sad condition had a higher learning rate than the happy, B = 11.86; and the data for distant influences were neutral between this theory and the null, B = 0.77. Thus, together the data for recent and distant influences provide strong support for the theory, overall B = 11.86*0.77 = 9.13. That is, the rate of implicit learning is sensitive to the use of mood inducing stimuli consistent with the predictions of the affect-as-information hypothesis (cf also [34]).

## Discussion

We introduce a method for measuring learning rate in a very simple prediction task. Its virtue is its simplicity and the transparency by which learning rate shows itself. The task is premised on implicit learning involving a strength of prediction of an event. Specifically, the method assumes that the strength of prediction on a given trial can be represented as a weighted mean of the strength on the previous trial and of what happened on the current trial. This assumption is a good characterisation of most models of implicit learning, including the Rescorla-Wagner rule in associative conditioning, error correction in connectionist networks, Kalman filters as used in models of reinforcement learning, or chunking models in which the strength of a chunk is incremented less as chunk strength approaches a ceiling [21], [35], [36]. The weighting for the current trial is the learning rate: The more the current trial is weighted, the more impact each trial has on changing the strength of prediction. The consequence of such a rule is that strength on a given trial is influenced by past trials in an exponentially decaying way. The slope of the decay is governed by learning rate: The larger the learning rate the stronger the influence of recent trials and the weaker the influence of past trials. This property of the learning rate can be used to measure it. To make measurement clear, we also used a random sequence so the influence of each trial on current predictions does not need to be adjusted by what happened at other time points; each trial is independent, thus making computations clean.

We show that people often develop expectations sensitive to events in the past; that is, there is learning. Further the phenomenology associated with this learning is largely that associated with implicit learning [16]–[18], [28], [37] (compare [38] for a similar task). Although people sometimes use conscious rules and recollections, they largely rely on guessing and intuition. Further, the learning people show is sensitive to context, consistent with it being associative.

We illustrate the usefulness of the method by showing it sheds light on important psychological questions. Paradoxically we argue that amnesia should be associated with a large learning rate in certain situations: A large learning rate means a small memory into the past and this is just what we find with a case study with a very dense amnesic. Future research could address the conditions under which amnesia is associated with an especially high or low learning rate on different versions of the task. According to the Bayesian approach, learning rates will be adjusted according to the probabilistic structure and dynamics of the domain (e.g. [39]). On this approach, people with amnesia may have trouble adjusting learning rates to deal with domains with learning rates that are optimally low (i.e. dealing with long time scales), but they will not in general be quicker than average to implicitly learn (for example, on a task that optimally has a relatively large learning rate). [40] found in an implicit spatial context learning task that presenting subjects first with a block of trials with no regularity to be learned inhibited subsequent learning of a regularity. Thus, using a genuinely random sequence may induce low learning rates in normal people on our task. Future research could explore if constantly changing the probability of the outcome, e.g. with a sinusoid, increases learning rate (note that the dependence between trials would then have to be partialed out to determine the profile of influence of past trials).

We use the affect-as-information hypothesis to predict that sad rather than happy moods should be associated with a large learning rate. Showing people sad faces as stimuli rather than happy ones indeed produced a larger learning rate. Future research could usefully explore the relation between emotion and learning rate. For example, can happy and sad images implicitly give success and failure feedback independently of mood? Conversely, is mood associated with a change in learning rate when the target stimuli are emotionally neutral? We hope these applications motivate other ideas in researchers. For example, we showed the importance of the valence of stimuli in affecting learning rate, but what about arousal, which we controlled? In general, how does the rate of learning depend on different contents? How does learning rate vary over time on a task or with different populations, or by drug induced changes to different neurotransmitter systems? 

A potential weakness of the method is that associative learning is not the only process that the task engages; people are also liable to the gambler’s fallacy [41], as is evident in Figures 1 and 2 for three time steps back, and four time steps back in the case of JC, where there is a tendency to predict the opposite to what happened. Overall, this influence is weak compared to the effect of implicit learning. In order to ensure the influence of the gambler’s fallacy was disentangled from implicit learning, trial-by-trial decisions were fit by a model consisting of a) a Rescorla-Wagner learning device with its learning rate, and simultaneously b) a gambler’s fallacy process with its equivalent rate parameter (see Materials and Methods). Controlling for gambler’s fallacy in this way, JC had an estimated learning rate of .80 (SD = .14), still detectably higher than that of controls (.63, SD = .32), t(60) = 3.01 (df adjusted for unequal variances), p = .004, d = 0.69. In fact, there was no detectable difference in gambler’s fallacy rate parameter between JC and controls (nor between people in the happy and sad conditions of the mood experiment). Nonetheless, future research could usefully explore conditions where the effect of the gambler’s fallacy could be mostly eliminated: For example, by using a situation more complex than a simple binary choice, by eliciting faster responses, or by using a cover story indicating the sequence was generated by human skill rather than a mechanical process [41].

The method assumes a learning process in which the enduring influence of a trial is determined completely by its contribution to a single overall strength term. But not all models of implicit learning make this assumption. For example, learning sequences of locations or musical tones has been successfully modelled with a “buffer network” in which the last n trials are explicitly represented and used to predict what happens on the next trial (i.e. there is a buffer of size n). [42]–[43]. In the models used by [42], [43], the stimuli up to n trials back would all have equal influence in prediction, and any stimulus more than n trials back would have no influence. While the buffer network (with n = 4) was successful in accounting for the relatively complex tasks of [42], [43], Figures 1, 2, 3, 4 show that the influence of past events was qualitatively different in the current task than the buffer model predicts. The buffer model could be made to fit the influence profiles shown in Figures 1, 2, 3, 4 by having a large buffer (up to at least n = 7) and adding an assumption that the representation of a stimulus decays according to n. This would ad hoc fit the data by brute force. The current method would then not so much measure learning rate as buffer size or the decay profile within the buffer. It would be measuring an interesting characteristic of the learning system, but not directly the learning rate of the component units in the network.

Another approach to modelling implicit learning that violates the assumptions of the method is the exemplar approach [44]–[46], in which correct responses are stored together with contexts. If a correct response together with general context (e.g. warning signal) was stored on each trial, there would be a flat influence profile back in time, as each time in the past would be equally represented. If these stored exemplars decayed, then most recent trials would have more influence, just as we find. In this case the learning rate measured by our method would reflect the decay rate of the exemplars.

Future research could examine our task in a more fine-grained way to determine if a model more complex than simple accumulating strength is needed to account for performance. Given the simplicity of the task and the fit of the profiles to such a simple model (barring the gambler’s fallacy effect), a Rescorla-Wagner model can be taken as at least an emergent approximation to the learning system, which defines a level of description to which we can assign a meaningful learning rate. It will be interesting to see how far such an approximation takes us, and what lower level features the measured learning rate reflects, for example, underlying learning rates of neurons.

Learning is a fundamental process characteristic of much of the brain; exploring the factors that the rate of learning depends on is hence a fundamental problem for psychology. It has been investigated in the animal learning domain, and its generalisation to people, with respect to particular problems, such as the relative learning rates of different stimuli of varying salience (over-shadowing) and effects of predictability or surprise on subsequent learning rate [47], [48]. Here we broaden the scope of the enquiry and provide a general tool for doing so. We show how exploring the problem of learning rate in people can produce interesting and surprising findings.

## Materials and Methods

### Bayesian analyses

A Bayes factor is useful for indicating continuous degrees of support for a hypothesis and hence when a null result counts against a theory that predicts a difference or doesn’t count one way or the other (see [49], [50]). Values around one indicate the data are equally consistent with both null and experimental hypotheses. Values greater than one indicate increasing evidence for the experimental hypothesis and values approaching zero indicate increasing evidence for the null. [51] regarded Bayes factors of greater than 3 or less than 1/3 as providing substantial evidence. A Bayes factor requires specification of what effect sizes the theory predicts. We based these predictions on a pilot study with 59 undergraduate students predicting whether a square will appear on the left or right. The mean degree of recent influences was .10, SD = .15, and of distant influences was .01, standard deviation  = .03 (both significantly above zero). For the mood study, the difference predicted by theory between happy and sad moods was modelled with half-normals with a standard deviations equal to the means for the pilot; i.e. .10 for recent influences and .01 for distant influences. That is, the theory was taken as predicting differences in the required direction on the order of magnitude of the obtained pilot means, with smaller differences being more likely than larger ones. See [49] and the associated website, http://www.lifesci.sussex.ac.uk/home/Zoltan_Dienes/inference/Bayes.htm, which provides explanation and an on-line Bayes factor calculator.

### Computational modelling

Trial-by-trial predictions were modelled with a Rescorla-Wagner learning device that predicted left or right based on one permanently on unit coding general context. It could have any learning rate between 0 and 1 in steps of 0.1. Specifically, let what happened on a trial, S, is coded one if the square was on the right and 0 if on the left; and the rate parameter be R and the current strength of prediction for ‘right’ being W, then the error in prediction was (S – l). W was updated according to: W = W + R*error.

A gambler’s fallacy process behaved in the same way except if the stimulus had just appeared on the left it increased the strength of prediction for right and vice versa; it thus also had a rate parameter between 0 and 1 in steps of 0.1. Specifically, let what happened on a trial, S, is coded one if the square was on the right and 0 if on the left; and the rate parameter be R and the current strength of prediction for ‘right’ being G, then G was updated according to: G = R*(1–S) + (1–R)*G.

On each trial the predictions of the two devices were combined with a weighted mean with a weight p for the Rescorla-Wagner output (and thus 1–p for the gambler’s fallacy) which also varied in steps of 0.1 between 0 and 1. That is, the overall strength of prediction for right was T = p*W + (1–p)*G. Thus the 300 trials of a given run of the experiment with a person was checked against the predictions of all 11 X 11 X 11 parameter combinations and the combination which minimised least mean square error was chosen as the best fitting parameter set. That is, error on a given trial was the difference between the subject’s response on that trial (coded 1 for right and 0 for left) and T. The error was squared and averaged over all 300 trials to provide a mean square error for a given model. Because the structure to be learned is random, and the Rescorla-Wagner and gambler fallacy processes are opposites, the error space is relatively flat around the minimum. Thus, this method of determining learning rate is less sensitive than the main method used in the text (i.e. directly testing differences in correlations). In other data we have found that the method becomes sensitive when the structure to be learned is non-random.

The estimated gambler’s fallacy rate for JC was .46 (SD = .40) and for normal controls .43 (SD = .35), not detectably different, t(66) = .28, 95% CI [–.17, .23]. Nonetheless, JC had an estimated learning rate of .80 (SD = .14), detectably higher than that of controls (.63, SD = .32), t(60) = 3.01 (df adjusted for unequal variances), p = .004, d = 0.69. For the mood study, people in the happy condition had an estimated gambler’s fallacy rate of .58 (SD = .32) not detectably different from those in the sad condition, .46 (SD = .37), t(54) = 1.24, 95% CI [–.07, .30]. Similarly, people in the happy condition had an estimated learning rate of .51 (SD = .34) not detectably different from the .63 (SD = .33) of people in the sad condition, t(54) = 1.34, though in the right direction and the confidence interval is wide in the predicted direction, [–.30, .06].
